# Cerebrospinal Fluid Drainage for Prevention of Spinal Cord Ischemia in Thoracic Endovascular Aortic Surgery—Pros and Cons

**DOI:** 10.1055/s-0042-1757792

**Published:** 2022-12-20

**Authors:** Hesham Ellauzi, Harendra Arora, John A. Elefteriades, Mohammad A. Zaffar, Rama Ellauzi, Wanda M. Popescu

**Affiliations:** 1 Aortic Institute at Yale New-Haven, Department of Cardiac Surgery, Yale University School of Medicine, New Haven, Connecticut; 2 Department of Surgery, Istishari Hospital, Amman, Jordan; 3 Department of Anesthesiology, University of North Carolina School of Medicine, Chapel Hill, North Carolina; 4 Department of Internal Medicine, Henry Ford Hospital, Detroit, Michigan.; 5 Department of Anesthesiology, Yale University School of Medicine, New Haven, Connecticut

**Keywords:** TEVAR, CSF drainage, spinal cord ischemia, spinal drain, cerebrospinal fluid

## Abstract

Thoracic endovascular aortic repair (TEVAR) carries a risk of spinal cord ischemia (SCI) which exerts a devastating impact on patient's quality of life and life expectancy. Although routine prophylactic cerebrospinal fluid (CSF) drainage is not unequivocally supported by current data, several studies have demonstrated favorable outcomes. Patients at high risk for SCI following TEVAR likely will benefit from prophylactic CSF drains. However, the intervention is not risk free, and thorough risk/benefit analysis should be individualized to each patient.

## Introduction

Thoracic endovascular aortic repair (TEVAR) is a minimally invasive procedure increasingly used in the treatment of thoracic aortic pathologies such as aneurysm, dissection, stenosis, and traumatic injury. The procedure was first reported by Volodos et al in 1987 1 and Dake et al 2 in 1994 for the repair of descending thoracic aortic aneurysms (TAAs) in patients at high surgical risk for conventional open repair. At the time of the inception of TEVAR, the risk of paraplegia for an open repair of thoracoabdominal aortic aneurysm was 16%. 3 TEVAR has been shown to reduce the risk of morbidity and mortality compared with conventional open surgical repair. 4 Patients with complex thoracic aortic pathologies who were previously deemed nonsurgical candidates for open surgical repair due to high surgical risk are now able to receive definitive treatment with endovascular stents. Despite TEVAR's favorable outcomes and steady advances in open surgical techniques, both endovascular and open surgical repairs still carry the potential risk of spinal cord ischemia (SCI) with subsequent paraplegia, a catastrophic complication.

### Spinal Cord Ischemia Effects on Life Expectancy

SCI exerts a devastating impact on patients in terms of quality of life and life expectancy. A retrospective review of 607 TEVAR patients revealed mean postoperative survival of 37.2 ± 4.5 months in patients who developed SCI, compared with 71.6 ± 3.9 months ( *p* < 0.0006) for those who did not develop SCI. Patients with SCI who manifested functional improvement showed much-improved survival of 53.9 ± 5.9 months compared with 9.6 ± 3.6 months for those with a permanent neurological deficit ( *p* < 0.0001). 5

To determine the impact of SCI on functional outcome and patient survival, Conrad et al performed a retrospective analysis on 576 patients undergoing open thoracoabdominal aneurysm repair, open descending thoracic aortic repair, and TEVAR. SCI was stratified by the degree of deficit and a scale was developed and graded as follows: grade I: flaccid paralysis; grade II: <50% function; and grade III: >50% function. A significantly higher 30-day mortality was registered in patients with SCI as compared with those without SCI, 23.4 versus 8%, respectively ( *p* < 0.001); 5-year survival of all SCI patients was less than half that of the non-SCI patients (25 ± 6 vs. 51 ± 3%, *p* < 0.001). Prognosis correlated closely with the degree of SCI deficit; patients with SCID grades II and III had similar 5-year survival to non-SCID patients (41 ± 10 vs. 51 ± 3%, *p* = 0.281). However, SCI deficit grade I conferred the worst prognosis, no patient recovered their ability to walk or lived to 5 years. Importantly, 73% of patients with SCI deficit grade II and 100% grade III patients were able to ambulate with or without assistance at their last follow-up visit. 6 Thus, the loss of the ability to walk correlated closely with mortality. It is, therefore, evident that the occurrence of SCI dramatically adversely impacts the overall quality of life and ultimately, life expectancy.

### Anatomy of Spinal Cord Blood Supply

The anterior spinal artery (ASA) provides the main blood supply to the spinal cord. The ASA supplies the anterior two-thirds of the spinal cord and is formed by the vertebral arteries, which in turn originate from the subclavian arteries. Before the vertebral arteries join to form the basilar artery, they branch inferiorly forming a single ASA. 7 The posterior one-third of the spinal cord is supplied by the two posterior spinal arteries (PSAs) which originate either from the posterior inferior cerebellar artery (PICA) or from the preatlantal vertebral arteries. 8

Through its course down the spinal cord, the ASA is augmented by an extensive collateral network. The segmental spinal arteries, intercostal and lumbar, bifurcate into an anterior and posterior radicular artery to feed both the ASAs and PSAs, respectively. The greater anterior radiculomedullary artery, also known as the artery of Adamkiewicz, is the largest segmental medullary artery that branches off the descending aorta and supplies the ASA. 8 Caudally, the hypogastric arteries provide retrograde collateral perfusion to the ASA. This extensive collateral network is crucial for preventing SCI when part of the spinal cord blood supply becomes compromised during an open or endovascular aortic repair.

### Spinal Cord Ischemia Risk Thoracic Endovascular Aortic Repair versus Open

**Fig. 1:**
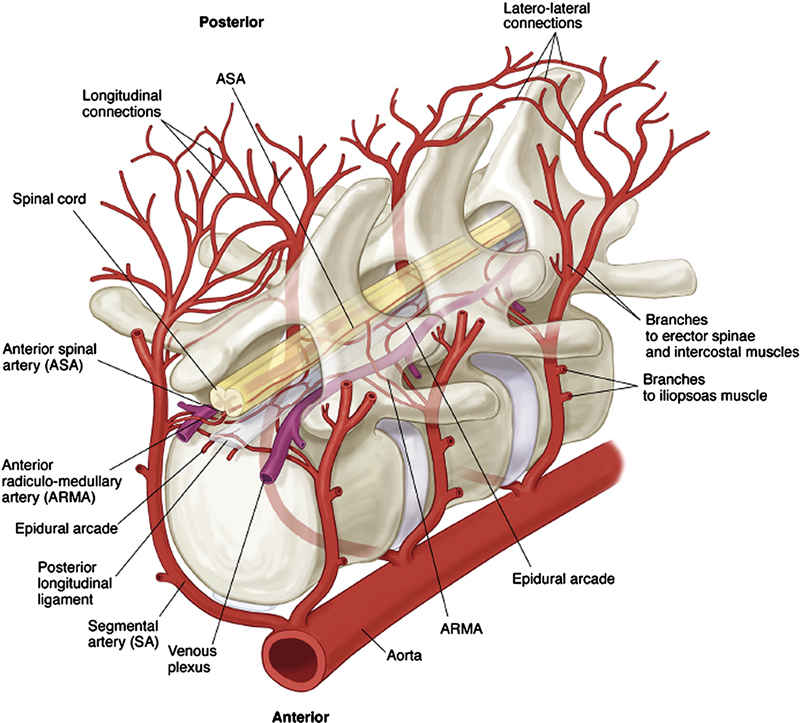
A diagrammatic reconstruction of blood supply to the spinal cord. The multiple inputs into the anterior spinal artery, and the rich matrix of longitudinal and lateral interconnections between the intraspinal and epidural systems are shown. ARMA, anterior radiculo-medullary artery; ASA, anterior spinal artery. (Reproduced with permission from: Etz et al 23 )

**Fig. 2:**
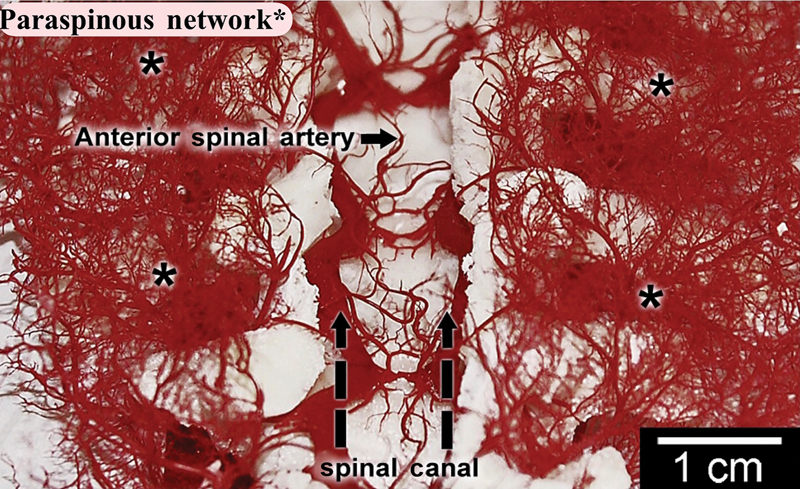
In this longitudinal section of a pig after injection of methyl methacrylate, the anterior spinal artery is seen, with its multiple connections with the extensive vasculature of the paraspinal muscles adjacent to the spinal cord (marked with asterisks). (Reproduced with permission from: Etz et al 23 )

A meta-analysis of 14,580 patients compared the complications encountered with TEVAR versus open repairs for descending thoracic aortic disease and found TEVAR to be associated with a lower incidence of postoperative paraplegia (3.3 vs. 5.5%, *p* = 0.007). 4 However, if patients with thoracoabdominal aortic disease were included and the extent of aortic coverage was comparable, the actual risk for SCI was similar between TEVAR and open repairs. Other studies have shown that the risk of SCI with TEVAR can reach 10.3%. 9 The risk of SCI following TEVAR varies and depends primarily on the extent of coverage of the segmental arteries ( Fig. 1 ) and the vigor of the paraspinal collateral network ( Fig. 2 ). Several risk factors predispose TEVAR patients to SCI. These include severe calcification or extensive coverage of the descending thoracic aorta (>15 cm), coverage of the left subclavian artery without revascularization, coverage of the celiac artery, or occlusion of the hypogastric plexus.

## Debate

Patients at high risk for SCI following TEVAR should receive special attention to optimize perfusion and oxygen delivery to the spinal cord. Some of the considerations for SCI monitoring and prevention include cerebrospinal fluid (CSF) drainage, blood pressure augmentation, neurophysiological monitoring, pharmacological adjuncts, and mild hypothermia. The current guidelines for patients with acute SCI recommend maintaining mean arterial pressure (MAP) above 85 to 90 mm Hg for 7 days following the injury. 10 This is based on the assumption that spinal cord perfusion pressure (SCPP) is equal to the MAP minus the CSF pressure or central vascular pressure, whichever is higher. Although this concept is extrapolated from the traumatic brain injury literature, with no direct supportive evidence, there is evidence supporting the concept that improving hemodynamics can enhance neurological outcomes. 11 12 In addition to blood pressure augmentation, SCPP can be optimized with CSF drainage by lowering the intrathecal pressure. CSF drains (CSFDs) have been extensively used for open thoracic and thoracoabdominal aortic surgeries, and this following discussion will focus on the ongoing debate whether prophylactic CSFDs should be routinely performed in patients undergoing high-risk TEVARs. Now that the above basic perspective has been explored, the debate is entered.

### Pros: For Spinal Cerebrospinal Fluid Drain

Recently, there has been increasing interest and debate regarding the prophylactic use of CSF drainage for the prevention of SCI. Although data supporting the benefit of lumbar CSFDs specifically in TEVAR are of moderate quality with no randomized controlled trials (RCTs), numerous studies have explored the utility of CSFD in open TAA repair. 13 14 A meta-analysis including three RCTs and five cohort studies (which compared outcomes after open thoracoabdominal aortic aneurysm repair to historical cohorts) showed a significant decrease in postoperative paraplegia with the use of lumbar CSF drainage, with a pooled odds ratio of 0.3 (number needed to treat [NNT] = 9, 95% confidence interval [CI], 0.17–0.54). 15

Several additional studies have assessed the use of CSFD in patients undergoing TEVAR and supported its role in SCI prophylaxis. A prospective observational trial performed by Hnath et al included 121 patients who underwent TEVAR. Fifty-six patients (46%) had prophylactic CSF drainage (notably this arm contained a higher proportion of patients at high risk for SCI—with more extensive aneurysmal coverage, previous abdominal aortic aneurysm repair, or coverage of the left subclavian artery), while the other 65 patients (54%) did not receive prophylactic drainage. Results showed that none of the patients with prophylactic CSFD developed SCI, while five patients (8%) in the control arm developed SCI within the first 24 hours of the procedure ( *p* = 0.05). 14 A more recently published study, which interrogated the database of the Vascular Quality Initiative, identified 4,287 patients who underwent TEVAR for various descending aortic pathologies. In the 1,292 propensity-matched pairs of patients, prophylactic placement of CSFD reduced the risk of SCI (1.5 vs 2.5%) but did not change the number of days spent in the ICU or the 30-day mortality rates. Additionally, this study identified that placement of a CSFD as a rescue maneuver did not offer the same degree of spinal cord protection as preoperative elective placement. 16

A 2016 meta-analysis included a total of 10 studies of both open and endovascular repairs of thoracoabdominal aortic aneurysm comparing 319 patients who received CSFD to 784 controls who did not. The study showed that CSFD decreased SCI by nearly half (relative risk of 0.42, 95% CI, 0.25–0.70; *p* = 0.0009), with an absolute risk reduction of 7% and the NNT to reach a benefit of 14. 13 In another study, Bobadilla et al observed 94 patients who underwent TEVAR with a mean length of aortic coverage of 16 cm. Their SCI protection protocol was routine CSF drainage (CSF pressure maintained < 10 mm Hg), endorphin receptor blockade (naloxone infusion), moderate intraoperative hypothermia (<35°C), and maintenance of MAP >90 mm Hg. Only one patient developed SCI postoperatively, who later recovered and was able to ambulate without assistance. 17

**Fig. 3:**
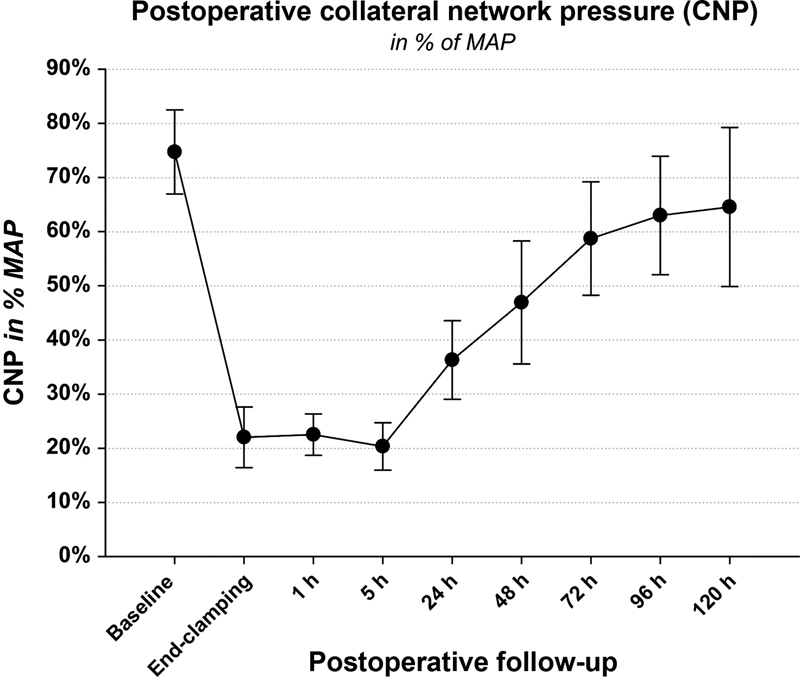
Collateral network pressure (CNP) measured in distal end of first lumbar segmental artery in 10 pigs after ligation of all segmental vessels. MAP, mean arterial pressure. (Reproduced with permission from Etz CD, Zoli S, Bischoff MS, Bodian C, Di Luozzo G, Griepp RB. Measuring the collateral network pressure to minimize paraplegia risk in thoracoabdominal aneurysm resection. J Thorac Cardiovasc Surg. 2010;140(6 suppl):S125)

**Fig. 4:**
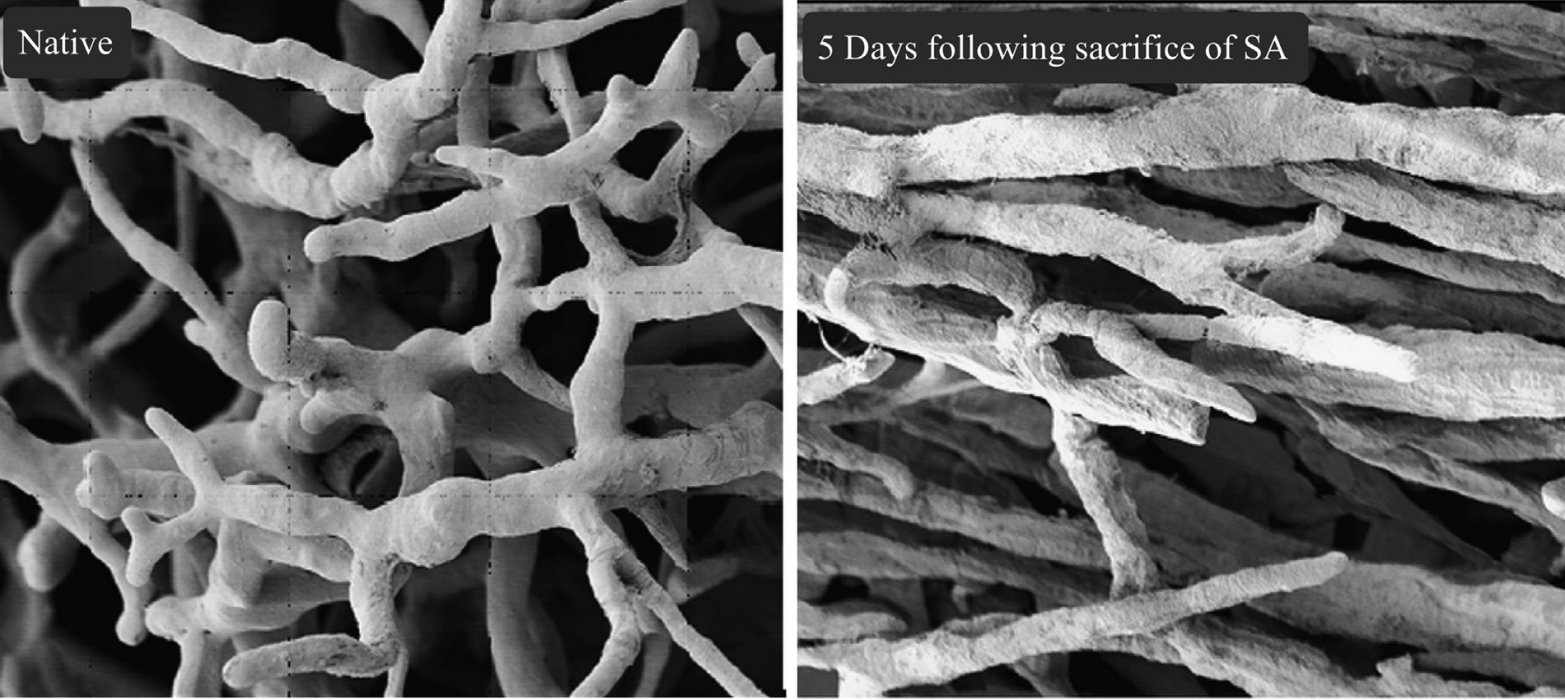
Paraspinous network vessel density increases 5 days following sacrifice of segmental arteries (SA), with significant realignment of paraspinous vessels from random distribution to parallel orientation along the spinal cord axis. (Reproduced with permission from: Etz et al 23 )

Furthermore, early experimental studies performed on animal models have demonstrated that CSF drainage reduces CSF pressure and mitigates the degree of SCI, improving neurological outcomes. 18 19 One such experiment, performed on 10 pigs, enhanced our understanding of the impact of sacrificing segmental arteries on the SCPP intraoperatively and in the immediate postoperative period. 20 Pigs have a spinal cord collateral blood supply similar to humans. 21 22 This experiment showed that at baseline the collateral spinal network pressure is 77% of the MAP. After clamping of segmental arteries, the collateral network pressure drops to 22 ± 6 mm Hg, reaching a nadir at 5 hours after the insult ( Fig. 3 ). Another experiment done on 10 Yorkshire pigs showed that 24 hours following extensive segmental artery sacrifice, the collateral network begins to recover by a process of angiogenesis and the pressure starts to increase, reaching approximately baseline levels at 5 days after the insult ( Fig. 4 ). 23 The study findings depicting an early collateral network deficiency, with subsequent recovery, suggest that early CSF drainage may be beneficial. In an endovascular procedure, the first few hours in the intensive care unit seem to be the most critical period when SCI can occur. Therefore, prophylactic CSF drainage along with blood pressure augmentation serves as a bridge until recovery of the collateral spinal network.

### American College of Cardiology Foundation and the American Heart Association Guidelines

In 2010, the American College of Cardiology Foundation and the American Heart Association (ACCF/AHA) assigned a class I recommendation for the use of CSFD as a protective strategy for the spinal cord in open and endovascular thoracic aortic repair, with level B evidence. 24 A class I recommendation indicates that the procedure is strongly recommended, beneficial, and should be performed. However, as the recommendation only pertained to those patients at high risk for SCI, risk stratification of patients undergoing TEVAR can be performed before the use of prophylactic CSF drainage. Risk factors associated with the development of SCI in patients undergoing TEVAR have been well characterized in previous studies 25 26 27 and include extensive aortic coverage (more than nine segments) by long aortic stent-grafts or multiple stent-grafts, sacrificing a greater number of segmental and intercostal arteries, coverage of the distal thoracic aorta (Th8–Th12), previous abdominal aortic aneurysm repair, 28 coverage of the left subclavian artery compromising collateral supply from the vertebral artery, 29 incomplete circle of Willis, chronic renal insufficiency, advanced atherosclerosis, 27 emergency operation, advanced age, and perioperative hypotension. Based on a retrospective evaluation of 7,900 patients undergoing various types of TEVAR procedures, with data obtained from the Society for Vascular Surgery/Vascular Quality Improvement Database, Mousa et al developed a preoperative risk stratification scale for SCI ( Table 1 ). Each risk factor is assigned several points, and patients are deemed to be at low, moderate, or high risk for SCI based on the total number of points accumulated. 30

**Table 1:** Risk scoring for spinal cord ischemia

**Variable**	**Points**
Age (by decade)	0.5
Celiac coverage	1
Current smoker	1
Dialysis	1.5
Three or more aortic devices	1
Emergent or urgent surgery	1
Adjunct procedures aorta related	1.5
Adjunct procedures not aorta related	1.5
Total device length 19–31 cm	1.5
Total device length ≥32 cm	3
ASA class 4 or class 5	1
Total procedure time ≥ 154 minutes	1
High volume center (50 or more)	−1
eGFR ≥ 60%	−1

Abbreviations: ASA, American Society of Anesthesiologists; eGFR, estimated glomerular filtration rate.

Notes: Categories of low, medium, or high risk: low risk = raw score 0 to 4; medium risk = raw score 4.5 to 6.5; high risk = raw score ≥ 7.0. Reproduced with permission from Mousa et al. 30

Level B evidence indicates moderate-quality evidence from one or more RCTs. Three RCTs have been published to date outlining the utility of prophylactic CSF drainage in open thoracoabdominal aortic aneurysm repair 31 32 33 ; however, no RCTs have yet been published on the endovascular approach.

Three articles have been taken into account in the class I recommendation for the use of CSF drainage as a protective strategy for the spinal cord in TEVAR for patients at high risk for SCI. The first study included 145 patients undergoing open thoracoabdominal aortic aneurysm repair and showed that CSFDs resulted in 80% reduction in the risk of postoperative neurological injury from SCI (2.6 vs. 13%, *p* = 0.03). 31 Although the study showed a significant reduction in neurological injury related to SCI, it only included open thoracoabdominal aortic aneurysm repair.

A meta-analysis performed by the Cochrane group, also considered for the class I recommendation, included three RCTs on open repairs and showed a significant reduction of SCI with the use of CSFD (odds ratio [OR] = 0.48; 95% CI, 0.25–0.92). However, one of the RCTs included in the meta-analysis always utilized intrathecal papaverine in combination with CSF drainage. If this study is excluded from the meta-analysis, the benefit of lumbar CSF drainage appears to be insignificant (OR = 0.57; 95% CI, 0.28–1.17). The authors' conclusion was that data supporting the role of CSFD in thoracic and thoracoabdominal aneurysm surgery for the prevention of neurological injury are limited. 34

The last study considered for the 2010 class I recommendation guideline was a retrospective cohort investigation analyzing 1,004 patients who underwent thoracic or thoracoabdominal open repairs over a 12-year period. Seven hundred and forty one patients (74%) had distal aortic perfusion (left atrial to femoral artery bypass) in combination with adjunctive CSFD, whereas the other 263 patients (26%) were treated in an earlier time period, with the traditional clamp-and-sew technique without either distal aortic perfusion or CSFD. The study compared these two groups and reported the SCI risk to be 2.4% in the CSFD adjunct group and 6.8% in the nonadjunct group ( *p* < 0.0009). Furthermore, the benefit was more evident in patients at high risk for SCI (patients with aneurysm distal to the left subclavian artery to below the renal arteries), with SCI occurring in 11 out of 167 (6.6%) in the adjunct group versus 11 out of 38 (29%) in the nonadjunct group. 35 However, no endovascular repairs were included in the study and the benefit may be attributed to the combination of both techniques, distal aortic perfusion and CSF drainage.

Based on this substantial but not ideal evidence, proponents strongly support the use of prophylactic CSFD in high-risk patients undergoing both open and endovascular thoracic aortic procedures.

### Cons: Against Spinal Cerebrospinal Fluid Drain

**Fig. 5:**
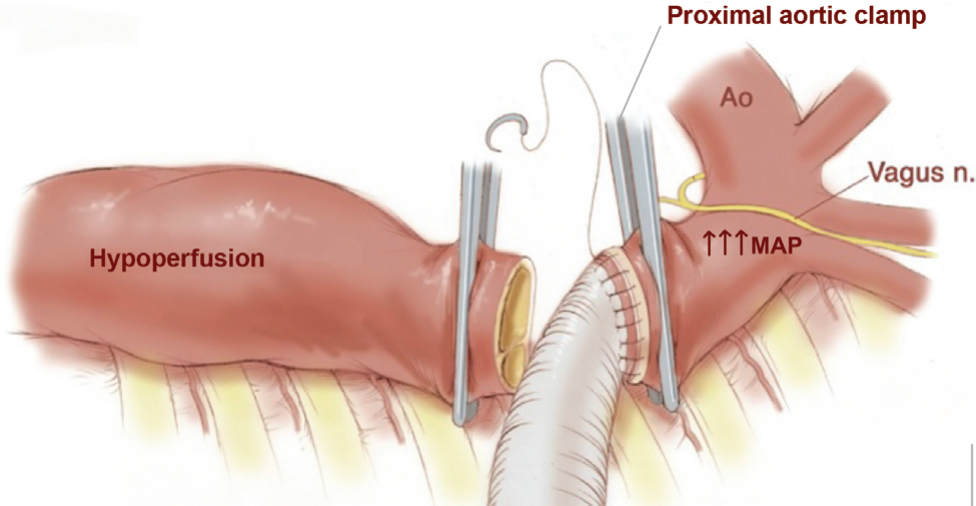
The use of aortic clamp in the historical clamp and sew technique in open aortic surgery causes a higher mean arterial pressure (MAP) proximal to the clamp and severe hypoperfusion distal to the aortic clamp. With the clamp-sew technique, there is no time for reimplantation of intercostal arteries, thus permanently depriving the spinal cord of collateral blood supply. (Reproduced with permission from Black JH, 3rd. Technique for repair of suprarenal and thoracoabdominal aortic aneurysms. J Vasc Surg. 2009;50:936–941)

It can be argued that results from open surgery may not translate to the endovascular approach as several distinct differences exist. For example, in open surgery, the historical clamp and sew technique ( Fig. 5 ) requires the use of an aortic clamp causing a higher MAP proximal to the clamp, with severe hypoperfusion distal to the aortic clamp. With the clamp and sew technique, there is no time for reimplantation of intercostal arteries, thus permanently depriving the spinal cord of collateral blood supply.

**Fig. 6:**
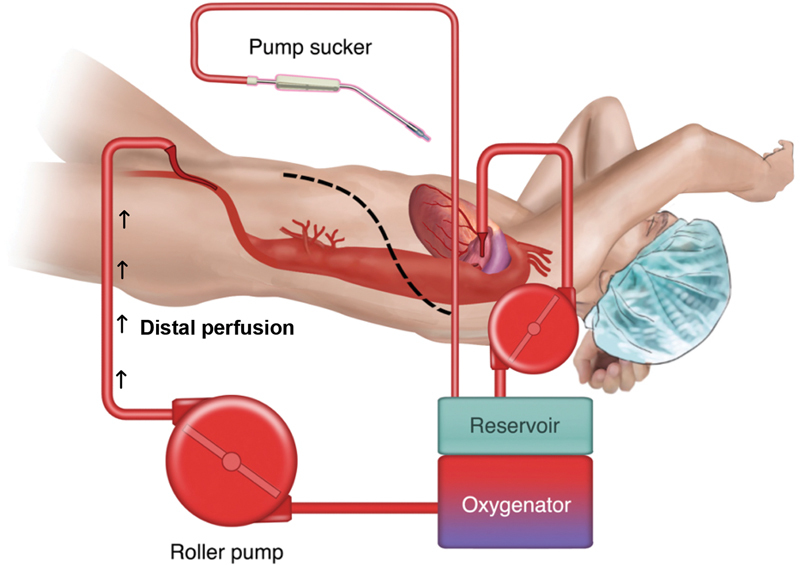
Standard left atrial-femoral bypass revolutionized open aortic repair by offering distal perfusion, which improves blood pressure below the clamp and allows time for segmental artery reimplantation. (Reproduced with permission from Papanikolaou D, Savio C, Zafar MA, et al. Left atrial to femoral artery full cardiopulmonary bypass: a novel technique for descending and thoracoabdominal aortic surgery. Int J Angiol. 2020;29:19–26)

**Fig. 7:**
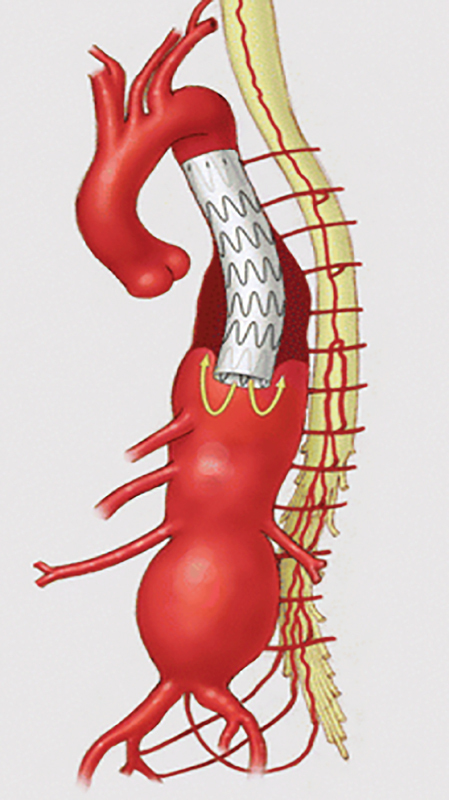
The graft body allows for continuous blood flow throughout endovascular repair procedure, resulting in much better hemodynamic stability and sustained lower body perfusion. (Reproduced with permission from Oderich GS, Baker AC, Banga P. Strategies to minimize risk of spinal cord injury during complex endovascular aortic repair. In: Oderich G. ed. In: Endovascular Aortic Repair. Cham, Switzerland: Springer; 2017:295–311)

Standard left atrial-femoral bypass ( Fig. 6 ) revolutionized open aortic repair by offering distal perfusion, which improves blood pressure below the clamp and allows time for segmental artery reimplantation, resulting in improved spinal cord perfusion. Similarly, in endovascular repair ( Fig. 7 ), the graft body allows for continuous blood flow throughout the procedure, resulting in much better hemodynamic stability and sustained lower body perfusion. Cardiovascular stability and the absence of aortic cross-clamping in the endovascular technique likely underlie the improved SCI outcomes, despite the inherent permanent sacrifice of segmental arteries. Experimental studies on sheep have shown that cross-clamping the aorta produces a much greater risk for SCI compared with covering the thoracic aorta with stent-grafts. 36

Moreover, also arguing against routine CSFD, some studies have shown that preoperative lumbar drainage is not associated with a reduced risk for SCI in TEVAR. 37 38 A recent systematic review by Wong et al identified 46 studies comprising a total of 4,936 patients, aiming to determine the appropriate role of CSFD in TEVAR. The overall incidence of SCI was reported to be 3.89%, and the pooled rate of SCI for patients who received prophylactic CSFD was 3.2% compared with 3.47% in patients who did not receive prophylactic CSFD. Furthermore, in 24 included studies, prophylactic CSFD was used selectively (for patients at high risk for SCI) and the pooled rate of SCI was 5.6%. The study concluded that the role of prophylactic CSFD in TEVAR is difficult to establish. 38

In a single-center study, which included 381 patients undergoing TEVAR over a period of 10-years, CSFD was utilized selectively based on SCI risk. Twenty-one percent (81/381) of the patients had preoperative CSFDs placed, of whom 14.8% (12/81) developed SCI; 6 of these were transient, whereas 6 resulted in permanent neurologic injury. In patients who did not receive a preoperative CSFD, SCI occurred in 4.3% (13/300) of the patients. In nine of these patients, SCI resolved with blood pressure augmentation. Three patients required late CSFD placement as SCI did not resolve with blood pressure augmentation alone, but they achieved complete resolution of symptoms. Only 1 of the 13 patients suffered permanent paraplegia. Moreover, in 32% (26/81) of the patients, preoperative CSFDs were never utilized as the CSF pressure never exceeded 12 mm Hg and none of these patients manifested signs of SCI. Drain-related complications occurred in 11% (9/81) of the patients, with no permanent injury registered. The study concluded that the utility of preoperative lumbar drains for the prevention of SCI during TEVAR procedures remains questionable. 37

### Cerebrospinal Fluid Drainage Complications and Safety Measures

As with any procedure, CSF drainage has its own complications, which vary in severity. These complications may be related to drain placement or removal, to CSF drainage per se, and to the indwelling spinal catheter. Some experts feel that prophylactic CSFD should be reserved for high-risk patients due to the significant risks associated with the procedure and that a thorough risk-benefit analysis should be performed before placing the CSFD. A recent retrospective single-center study evaluated the utility of CSFDs in preventing SCI and the frequency of drain-related complications. Consistent with previous literature, this study identified that the group that had a prophylactic CSF drainage had a lower incidence of SCI (1.2 vs. 2.9%), but the 30-day mortality was similar in both groups. However, the study also identified a 6% risk of CSFD-related complications, none of which were permanent. 39 Another very recent study that assessed the safety of perioperative CSFD placement came to similar conclusions. 40 A large meta-analysis performed to define a more accurate risk-benefit ratio analyzed 34 studies from 1990 to 2017 and included 4,714 patients who had CSFD placed for open or endovascular repairs of thoracic or thoracoabdominal aortic aneurysms. The study identified the overall CSFD-related complication rate at 6.5%, with severe complications occurring in 2.5% of the cases and a pooled CSFD-related mortality rate of 0.9%. 41 Severe complications included subarachnoid or intracranial hemorrhage (ICH), epidural hematoma, meningitis, and drainage or catheter-related neurological deficit. Therefore, the placement of a CSFD is not without risk and should be heavily weighed against the benefit.

A retrospective review performed by Wynn et al studied complications of CSFD performed in 724 patients. A total of 10% of the patients developed bloody CSF, of whom half had an ICH diagnosed on computed tomography (CT) of the brain. The general interpretation is that dropping CSF pressure in the spinal canal can lead to the caudal anatomic displacement of the brain, stretching fragile veins and leading to bloody CSF. Of those patients with ICH, six (15%) were symptomatic and three (7.5%) died. These authors further reported that a higher volume of CSF drainage correlated with the likelihood of intracerebral bleeding and, therefore, suggested that the amount of CSF drainage should be restricted to no more than 10 to 20 mL per hour along with a CSF pressure target of 8 to 10 mm Hg. 42 As identified by this study, mortality related to CSFD is highest in patients with symptomatic ICH. Patients with bloody CSF, unexplained headache, decreased level of consciousness, or a neurological deficit not related to SCI should undergo a head CT emergently to rule out ICH. The risk of ICH related to CSF drainage can be minimized by intermittently draining 10 to 20 mL per hour and maintaining an ICP between 8 and 10 mm Hg (in the absence of SCI).

Although CSF drainage carries a risk of serious complications, adherence to safety measures can significantly minimize this risk. Authorities point to methods of risk reduction, including fluoroscopy-guided CSFD placement, avoidance of over-drainage of CSF, placement of drains a day prior to surgery, and protocol-based management of CSFDs. Some studies have reported no cases of spinal or epidural hematomas. 43 44 Less serious CSFD complications have been shown to occur at a rate of 2 to 3.7% and include puncture site bleeding, CSF leak, hypotension, spinal headache, and catheter fracture. 41 Another study performed to evaluate the safety of CSFD in 135 patients who underwent open thoracic aortic surgery using extracorporeal circulation and full anticoagulation did not result in any hemorrhagic complications, with the most frequent complication being catheter fracture at 1.8%, 43 which can be minimized with the proper surgical technique.

Thus, opponents of spinal drainage have meaningful data on which to draw, data that confirms rare but real potential complications and equivocal benefits.

## Conclusion

There is tangible, credible evidence to support both sides of the debate—as is seen so often, because medicine, even at the level of complex surgical procedures, remains an art rather than true science.

Even though endovascular repair has a lower risk than open thoracoabdominal aortic aneurysm surgery, SCI with potentially permanent paraplegia remains a possible complication of TEVAR. CSF drainage allows for SCPP optimization during critical periods of hypoperfusion. According to the ACCF/AHA guidelines, CSFD is recommended for patients at high risk for SCI undergoing TEVAR procedures. Although there are no RCTs to validate the utility of preoperative CSFD specifically in TEVAR procedures, several recent studies have demonstrated favorable outcomes. 13 14 17 45 46 Nevertheless, routine prophylactic CSF drainage is not unequivocally supported by current data. In conclusion, the significant economic burden associated with morbidity and mortality of SCI, the tragic consequences of rescue drainage failure, combined with the low risks for CSF drainage complications, in our view justify the use of prophylactic CSFDs in high-risk patients undergoing open surgical repair or TEVAR. 47 However, the intervention is not risk-free, and a thorough risk/benefit analysis should be individualized to each patient.
